# The Prevalence of Potential Drug-Drug-Gene Interactions: A Descriptive Study Using Swiss Claims Data

**DOI:** 10.2147/PGPM.S527556

**Published:** 2025-08-23

**Authors:** Nina L Wittwer, Christoph R Meier, Carola A Huber, Julie D Moser, Henriette E Meyer zu Schwabedissen, Samuel S Allemann, Cornelia Schneider

**Affiliations:** 1 Basel Pharmacoepidemiology Unit, Division of Clinical Pharmacy and Epidemiology, Department of Pharmaceutical Sciences, University of Basel, Basel, Switzerland; 2 Hospital Pharmacy, University Hospital Basel, Basel, Switzerland; 3 Boston Collaborative Drug Surveillance Program, Lexington, MA, USA; 4 Department of Health Sciences, Helsana Group, Zürich, Switzerland; 5 Biopharmacy, Department of Pharmaceutical Sciences, University of Basel, Basel, Switzerland; 6 Pharmaceutical Care, Department of Pharmaceutical Sciences, University of Basel, Basel, Switzerland

**Keywords:** drug-drug interaction, drug-drug-gene interaction, phenoconversion

## Abstract

**Purpose:**

We aimed to determine the prevalence of interactions between PGx drugs metabolized by CYP2C9, CYP2C19, and CYP2D6 and drugs that act as inhibitors or inducers of those enzymes in the Swiss population.

**Patients and Methods:**

We defined concomitant use of PGx drugs and inhibitors/inducers as instances where a claim of a PGx drug and a claim of an inducer or inhibitor concerning the same enzyme were made within a specified temporal window, either ± 5 days or ± 30 days. We assessed concomitant drug use between 2017 and 2021, using claims data from a Swiss insurance company (Helsana).

**Results:**

Out of 894,748 individuals continuously insured, between 17.4% (± 5-days window) and 24.8% (± 30-days window) were exposed to potentially interacting drug pairs, with 1.5% to 2.2% being exposed to potentially strong interacting drug pairs. Individuals exposed to potentially interacting drugs were more frequently female, older and took a greater number of drugs than the general population. The majority of potential interactions were associated with CYP2D6 or CYP2C19.

**Conclusion:**

In light of the high prevalence of the simultaneous use of PGx drugs with inhibitor and inducer drugs, it is imperative to consider non-genetic factors, such as drug-induced phenoconversions, when interpreting PGx test results.

## Introduction

The effectiveness of drug therapies is influenced by a number of factors, including the concomitant use of other drugs, nutritional status, age, sex, disease status and genetics.^1^,^2^ Genetic variations have the potential to modify the pharmacokinetics and pharmacodynamics of a drug. In severe cases, this may impact the safety and effectiveness of a drug.^3–5^ A drug therapy should therefore ideally be personalized to each individual patient, also taking into account an individuals’ genetics.^6^ It has been demonstrated that preemptive pharmacogenetic (PGx) testing can reduce the incidence of adverse drug reactions (ADRs).^5^ Another common cause of ADRs are drug-drug interactions (DDIs).^7–10^ However, while DDIs are often evaluated in clinical practice, drug-gene interactions (DGIs) and drug-drug-gene interactions (DDGIs) are not.^11^

Determining the genotype of an individual can be applied to predict the metabolization of active ingredients (phenotype). If the actual phenotype deviates from the genotype-predicted phenotype due to non-genetic factors, this is referred to as phenoconversion.^12^ These non-genetic factors include disease-related factors (eg cancer), lifestyle-related factors (eg weight, smoking) or physical factors (eg age, pregnancy). Phenoconversions can also be linked to concomitantly used drugs, and are then referred to as DDGIs.^12–14^ For example, phenoconversions into a slower metabolism phenotype can occur with concomitant cytochrome P450 (CYP) inhibitors, increasing age, cancer, and inflammation, whereas concomitant use of CYP450 inducers and smoking may result in a faster metabolism phenotype.^12^ However, the effect depends on the genetic predisposition as well as on the strength of the inhibitor or inducer.^12^,^15^ For example, if a CYP2D6 poor metabolizer (PM) is treated with a CYP2D6 inhibitor, the individual will remain a CYP2D6 PM, as there is no enzyme to inhibit.^12^ Phenoconversions have significant implications for the analysis and interpretation of genotype-based association studies of clinical outcomes and ultimately for the personalization of therapy in everyday clinical practice.^3^ Phenoconversions are neglected in both clinical practice and research, even though they are a frequent phenomenon.^12^,^16^ Mostafa et al investigated phenoconversions in an Australian cohort and found a fivefold increase in PMs of CYP2C19 and CYP2D6 due to concomitant use of inhibitors or inducers and therefore drug induced phenoconversions.^2^ A Swiss study compared phenotyping results and predicted phenotypes in a small group of patients taking antidepressants. They found discrepancies between the predicted metabolic status and the actually measured status in 33–65% of the patients.^17^ This suggests that phenoconversion is a common phenomenon.

Some advocate the use of therapeutic drug monitoring (TDM), as a tool used to prevent ADRs associated with DDIs. However, TDM is not useful to prevent ADRs at the start of therapy, as this can only be done once steady state has been reached.^3^ As information on an individual’s phenotype is more frequently available in clinical routine, PGx testing incorporating DDGIs may be a more effective approach.^18^ Guidelines are essential for the implementation of PGx into clinical practice.^3^ So far, guidelines for over 100 drug-gene pairs exist and support healthcare professionals in deciding how and whether a dosage should be adjusted based on a genetic variant, or whether an alternative active substance should be used.^19^ These guidelines are published by working groups such as the CPIC (Clinical Pharmacogenetics Implementation Consortium),^20^ or the DPWG (Dutch Pharmacogenetics Working Group).^21^ However, explicit recommendations for the management of DDGIs are still sparse.

Not accounting for drug-induced phenoconversion may undermine the effectiveness of PGx interventions in determining the correct dosage for individual patients. While these important limitations of PGx-guided prescribing are widely recognized, the true extent of drug-induced phenoconversion remains largely unclear. The aim of this study was to determine the frequency of interactions between PGx drugs and drugs that act as inhibitors or inducers of the enzyme or transporter in question in the Swiss population. We therefore examined claims data for such concomitant use, and analyzed which drugs were involved in the interactions and characterized the individuals affected.

## Materials and Methods

### Data Source

We analyzed data from the Helsana database, a claims database comprising data from the Helsana Group, a Swiss health insurance company. Approximately 1.2 million individuals are covered by Helsana’s basic healthcare insurance, which represents approximately 14% of the Swiss population.^22^ In Switzerland, the provision of basic health insurance is mandatory for all residents. In addition, supplementary insurance can be purchased at the discretion of the individual. Swiss health insurance companies are privately owned and can be freely selected by everyone, with the option to change providers annually. Health insurance companies are prohibited from denying any individual coverage for basic insurance.^23^ Consequently, the data generated by Swiss health insurance companies is considered comprehensive with regard to different age groups, cantons of residence, and sex. The benefits of basic insurance are determined by the Federal Office of Public Health (FOPH) and include, among other things, all medicines on the specialties list.^24^

The Helsana claims database records electronically submitted claims by service providers. The database records information on medication purchases from the outpatient sector including the date of purchase, the medication name, and the package size, using the Anatomical Therapeutic Chemical Classification System (ATC). Furthermore, the Helsana database incorporates patient demographic data including the patient’s birth year, canton of residence, and sex. It should be noted that medications dispensed during hospitalization are not covered, except for those deemed to be highly expensive, as they are billed using a prospective payment system based on diagnosis-related groups (DRGs).^25^ Furthermore, the purchases of drugs without a prescription (over-the-counter, OTC) are not fully recorded. Furthermore, the Helsana database does not contain information on lifestyle factors (eg smoking status or weight), outpatient diagnoses, laboratory test results or genetic information.

Numerous studies on the use and safety of medicines published in peer-reviewed journals have been based on the Helsana database.^26^

### Substrates, Inhibitors, and Inducers

In this study, we restricted our evaluation to concomitant drug claims involving the enzymes CYP2C9, CYP2C19, and CYP2D6. The three enzymes were selected for analysis because previous studies have demonstrated that these are the most relevant pharmacogenes, as approximately 80% of all DGIs are caused by them.^5^,^27^ For patients claiming PGx drugs associated with CYP2C9, CYP2C19, or CYP2D6, each inhibitor or inducer of these enzymes was identified.

PGx drugs were defined as drugs with a PGx guideline with recommendations associated with CYP2C9, CYP2C19, or CYP2D6 on the Pharmacogenetic Knowledgebase (PharmGKB) on 25 January 2024.^19^ This resulted in 51 PGx drugs (14 substances were defined as CYP2C19 substrates, 14 as CYP2C9 substrates, and 28 substances as substrates of CYP2D6). We classified drugs as inhibitors or inducers based on the Flockhart table,^28^ the US Food and Drug Administration (FDA) drug list,^29^ the interaction table of the Hospital of Geneva,^30^ and the DrugBank online.^31^ Only inducers that were categorized as either “strong” or “moderate” were included in the study, as these are regarded as clinically relevant.^3^ If the databases had a differing classification, the respective substances were classified as moderate, to prevent overestimation of their effect. Furthermore, only inhibitors and inducers categorized based on in vivo data were included in the analysis. The analysis was limited to guidelines for individual substances, given the substance specificity of PGx. Additionally, five substances (ecstasy, curcuma/curcumin, rhein, ritanserin and the combination of tipranavir with ritonavir) were excluded due to the absence of available ATC codes. In total, 37 substances were defined as CYP2C19 inhibitors, 42 as CYP2C9 inhibitors, and 78 substances as inhibitors of CYP2D6. Furthermore, 16 substances were defined as CYP2C19 inducers, 12 as CYP2C9 inducers, and no substances as inducers of CYP2D6. All substances were categorized according to their ATC code as either locally or systemically administered drugs. An ATC code encompassing both applications was classified as systemically administered. Table 1 presents a comprehensive overview of all included substrates, inhibitors, and inducers. Further details on the classification of ATC codes can be found in the Supplementary Material (Tables S1–S3).

**Table 1:** Substrates, Inhibitors, and Inducers

Gene			Drugs
**CYP2C19**	Substrates		Amitriptyline, citalopram, clomipramine, clopidogrel, dexlansoprazole, doxepin, escitalopram, imipramine, lansoprazole, omeprazole, pantoprazole, sertraline, trimipramine, voriconazole
	Inhibitors	Strong	Chloramphenicol, clobazam, clomipramine, delavirdine, fluvoxamine, gemfibrozil, imipramine, lansoprazole, miconazole, modafinil, stiripentol, tioconazole
		Moderate	Abiraterone, armodafinil, cenobamate, cisapride, clopidogrel, desogestrel, efavirenz, eslicarbazapeine acetate, esomeprazole, etravirine, felbamate, fluconazole, fluoxetine, gefitinib, isoniazid, ketoconazole, moclobemide, omeprazole, oxcarbazepine, quetiapine, sertraline, ticlopidine, topiramate, voriconazole, zafirlukast, zucapsaicin
	Inducers	Strong	Phenobarbital, primidone, rifamycin, rifapentine, rifaximin, ritonavir
		Moderate	apalutamide, carbamazepine, dexamethasone, efavirenz, enzalutamide, letermovir, phenytoin, rifabutin, rifampicin, St. John’s wort
**CYP2C9**	Substrates		Acenocoumarol, celecoxib, fluindione, flurbiprofen, fluvastatin, fosphenytoin, ibuprofen, lornoxicam, meloxicam, phenytoin, piroxicam, siponimod, tenoxicam, warfarin
	Inhibitors	Strong	Capecitabine, delavirdine, etravirine, floxuridine, fluvastatin, gemfibrozil, nicardipine, silibinin, sulfaphenazole, tepotinib, voriconazole
		Moderate	Abiraterone, amiodarone, atazanavir, chloroquine, clopidogrel, clotrimazole, crisaborole, efavirenz, felbamate, fluconazole, fluoxetine, fluvoxamine, imatinib, iproniazid, irbesartan, ketoconazole, losartan, medical cannabis, metronidazole, miconazole, mifepristone, nabilone, nateglinide, phenylbutazone, quetiapine, quinidine, sorafenib, sulfamethoxazole, sulfinpyrazone, troglitazone, valproic acid
	Inducers	Strong	Dabrafenib, phenobarbital, phenytoin, primidone
		Moderate	Alpelisib, bosentan, carbamazepine, dexamethasone, elvitegravir, enzalutamide, rifampicin, ritonavir
**CYP2D6**	Substrates		Amitriptyline, aripiprazole, atomoxetine, brexipiprazole, clomipramine, codeine, desipramine, doxepin, eliglustat, flecainide, fluvoxamine, haloperidol, hydrocodone, imipramine, metoprolol, nortriptyline, ondansetron, paroxetine, pimozide, propafenone, risperidone, tamoxifen, tramadol, trimipramine, tropisetron, venlafaxine, vortioxetine, zuclopenthixol
	Inhibitors	Strong	Amiodarone, bupropion, cisapride, clomipramine, dacomitinib, flecainide, fluoxetine, glycerol phenylbutyrate, haloperidol, imatinib, levomepromazine, midostaurin, orphenadrine, paroxetine, promethazine, propafenone, quinidine, thioridazine
		Moderate	Abiraterone, asunaprevir, berotralstat, celecoxib, chloroquine, chlorpromazine, ciclosporin, cimetidine, cinacalcet, citalopram, clobazam, clotrimazole, clozapine, cholecalciferol, darifenacin, delavirdine, desipramine, diphenhydramine, dosulepin, doxepin, dronedarone, duloxetine, fluvoxamine, fusidic acid, gefitinib, halofantrine, imipramine, ketoconazole, lercanidipine, lorcaserin, lumefantrine, manidipine, methadone, metoclopramide, metoprolol, mirabegron, moclobemide, nicardipine, nilotinib, panobinostat, perhexiline, pitolisant, primaquine, quetiapine, quinine, risperidone, ritonavir, rolapitant, rucaparib, sertraline, sorafenib, sulconazole, sulfaphenazole, tegaserod, terbinafine, terfenadine, tipranavir, tranylcypromine, venlafaxine, vilazodone

### Statistical Analysis

We conducted a retrospective, descriptive study with data encompassing the period from 1 January 2017 to 31 December 2021. The study included all individuals who were continuously insured with basic health insurance at Helsana from 2017 to 2021. The age of an individual was calculated at the end of 2017.

In this study, concomitant use of PGx drugs and inhibitors/inducers was defined as instances in which a PGx drug and an inducer or inhibitor of the same enzyme were claimed within a certain time frame (± 5 days or ± 30 days). In instances where an individual exhibits multiple potentially interacting drug pairs associated with the same enzyme, they are considered only once. Agents that were classified both as a substrate and an inducer/inhibitor cannot enter an interaction with themselves.

We assessed the concomitant use of PGx drugs and inhibitors/ inducers over the five-year period (2017–2021) stratified by sex. We quantified the number of individuals affected by concomitant use and assessed the most frequently potentially interacting drug pairs.

We performed the statistical analyses using SAS 9.4 software (SAS Institute INC., Cary, NC) and Excel for Microsoft 365 (version 2308).

### Ethics Approval

According to Article 22 of the Swiss Data Protection Act, no ethics approval is required for retrospective studies with anonymized data.^32^

## Results

### Study Population

In total, 894,748 individuals have made 71,451,678 drug claims between the years 2017 and 2021. Most individuals (70.3%) claimed at least one PGx drug, while 62.1% claimed a CYP inhibitor and 24.7% an inducer, respectively. The mean age of the individuals was 44.5 ± 24.0 years, with an average of 19.7 ± 16.7 different drugs claimed over the five-year period. Women were more frequently exposed to PGx drugs, inhibitors, and inducers, claimed on average more different drugs, and were on average older than men (Table 2).

**Table 2:** Characteristics of the Study Population

	Total	Men	Women
N (%)			
**Insured Individuals**	894,748 (100)	425,852 (47.6)	468,896 (52.4)
**With Drug Claims**	850,844 (95.1)	396,098 (46.6)	454,746 (53.4)
**With Claims of PGx Drugs**			
Any PGx Drug	628,878 (70.3)	279,932 (44.5)	348,946 (55.5)
Systemic PGx Drugs	628,852 (70.3)	279,920 (44.5)	348,932 (55.5)
Associated with CYP2C19	381,490 (42.6)	160,651 (42.1)	220,839 (57.9)
Associated with CYP2C9	450,610 (50.4)	194,545 (43.2)	256,065 (56.8)
Associated with CYP2D6	277,741 (31.0)	116,590 (42.0)	161,151 (58.0)
**With Claims of Inhibitors**			
Associated with CYP2C19	289,533 (32.4)	116,755 (40.3)	172,778 (59.7)
Associated with CYP2C9	272,275 (30.4)	100,554 (36.9)	171,721 (63.1)
Associated with CYP2D6	471,774 (52.7)	189,938 (40.3)	281,836 (59.7)
**With Claims of Inducers**			
Associated with CYP2C19	220,830 (24.7)	86,499 (39.2)	134,331 (60.8)
Associated with CYP2C9	205,198 (22.9)	81,919 (39.9)	123,279 (60.1)
**Mean per Individual ± sd**			
**Drugs**	19.7 ± 16.7	17.8 ± 14.9	23.3 ± 17.4
**Age [Years]**	44.5 ± 24.0	42.7 ± 23.5	46.1 ± 24.3

**Abbreviations**: N: number of individuals, PGx: pharmacogenetic, sd: standard deviation, %: percentage of the category “total” is calculated in relation to the total number of insured individuals, %: percentage of the category “men” and “women” is calculated in relation to the “total”.

### Concomitantly Used Drug Pairs

A total of 15,594,909 concomitant drug claims, involving a PGx drug and inducer or inhibitor, were registered between 2017 and 2021, using the ±30-days window. A total of 3,415,833 concomitant drug pair claims were registered using the ±5-days window. When limiting the interactions to strong inhibitor and inducers and systematically administered drugs, 482,732 concomitant drug pair claims were registered during the ±30-days window, or 126,861 concomitant drug pair claims were registered during the ±5-days window. In total, 24.8% (±30-days window) or 17.4% (±5-days window) of individuals were exposed to concomitantly used drug pairs (see Table 3). If only strong and systemic acting drugs were included, 1.5% (±5-days window) or 2.2% (±30-days window) of individuals were exposed to concomitantly used drug pairs. Between 24.8% (±5-days window) and 35.3% (±30-days window) of PGx drug users used concomitantly claimed inhibitors/ inducers. In the ±30-days window, the highest percentage of individuals were exposed to concomitantly used drug pairs associated with CYP2C19. In contrast, in the ±5-days window, the highest percentage of individuals was exposed to concomitantly used drug pairs associated with CYP2D6. CYP2C9 was associated with concomitantly used drug pairs the least frequently during both time frames. Women were more frequently exposed to concomitantly used drug pairs than men. The mean age of individuals with interactions in the ±5-days window was 58.7 ± 21.1 years, with an average of 37.0 ± 18.1 drugs claimed. Individuals with interactions in the ±30-days window had a mean age of 57.8 ± 21.1 years and took on average 38.6 ± 18.9 drugs.

**Table 3:** Individuals with Concomitantly Used Drug Pairs Stratified by Sex and Temporal Window

Enzyme	Interaction Type	Claim of Drug Pairs Within ± 5 Days			Claim of Drug Pairs Within ± 30 Days		
		Total n=894,748	Men n=425,852	Women n=468,896	Total n=894,748	Men n=425,852	Women n=468,896
		N (%)					
**Any**	Any	155,934 (17.4)	59,106 (13.9)	96,828 (20.7)	222,285 (24.8)	84,418 (19.8)	137,867 (29.4)
	Systemic	139,629 (15.6)	52,217 (12.3)	87,412 (18.6)	194,489 (21.7)	72,669 (17.1)	121,820 (26.0)
	Strong	13,918 (1.6)	5526 (1.3)	8392 (1.8)	21,994 (2.5)	8620 (2.0)	13,374 (2.9)
	Strong and Systemic	13,034 (1.5)	5200 (1.2)	7834 (1.7)	20,077 (2.2)	7909 (1.9)	12,168 (2.6)
**CYP2C19**	Any	74,860 (8.4)	29,358 (6.9)	45,502 (9.7)	122,872 (13.7)	46,934 (11.0)	75,938 (16.2)
	Systemic	64,979 (7.3)	25,337 (5.9)	39,642 (8.5)	103,545 (11.6)	39,151 (9.2)	64,394 (13.7)
	Strong	3529 (0.4)	1393 (0.3)	2136 (0.5)	6478 (0.7)	2531 (0.6)	3947 (0.8)
	Strong and Systemic	2582 (0.3)	1048 (0.2)	1534 (0.3)	4390 (0.5)	1770 (0.4)	2620 (0.6)
**CYP2C9**	Any	52,077 (5.8)	18,248 (4.3)	33,829 (7.2)	87,639 (9.8)	30,517 (7.2)	57,122 (12.2)
	Systemic	42,193 (4.7)	14,641 (3.4)	27,552 (5.9)	67,957 (7.6)	23,426 (5.5)	44,531 (9.5)
	Strong	764 (0.1)	317 (0.1)	447 (0.1)	1500 (0.2)	629 (0.1)	871 (0.2)
	Strong and Systemic	764 (0.1)	317 (0.1)	447 (0.1)	1500 (0.2)	629 (0.1)	871 (0.2)
**CYP2D6**	Any	78,253 (8.7)	28,219 (6.6)	50,034 (10.7)	110,943 (12.4)	40,336 (9.5)	70,607 (15.1)
	Systemic	74,119 (8.3)	26,287 (6.2)	47,832 (10.2)	103,489 (11.6)	36,952 (8.7)	66,537 (14.2)
	Strong	10,160 (1.1)	4009 (0.9)	6151 (1.3)	15,186 (1.7)	5889 (1.4)	9297 (2.0)
	Strong and Systemic	10,160 (1.1)	4009 (0.9)	6151 (1.3)	15,186 (1.7)	5889 (1.4)	9297 (2.0)

**Abbreviations**: N: Number of individuals, %: percentage is calculated in relation to the total number of individuals.

The drug pairs most frequently, concomitantly used were pantoprazole-quetiapine, citalopram-quetiapine, and metoprolol-quetiapine during the ±30-days window (Table 4). During the ±5-days window, the most frequently concomitantly used drug pairs were pantoprazole-quetiapine, escitalopram-quetiapine, and citalopram-quetiapine (Table 5). The top 15 drug pairs consisted of moderate inhibitions of CYP2C19 and CYP2D6. The most frequently observed strong interaction was between trimipramine and haloperidol, with 40’532 claims during the ±30-days window. During the ±5-days window it was between metoprolol and amiodarone, with 10,198 claims. The complete list of potentially interacting drug pairs can be found in Tables S4 and S5.

**Table 4:** Top 15 Most Claimed Pairs of PGx Drugs and Inhibitors or Inducers Within ± 30 Days in the Five-Year Observation Period From 2017 to 2021

PGx drug	Inhibitor/ Inducer	Interaction Type	Enzyme	Number of Concomitant Claims
Pantoprazole	Quetiapine	Moderate inhibition	CYP2C19	2,767,924
Citalopram	Quetiapine	Moderate inhibition	CYP2C19	701,040
Metoprolol	Quetiapine	Moderate inhibition	CYP2D6	683,822
Escitalopram	Quetiapine	Moderate inhibition	CYP2C19	662,628
Haloperidol	Quetiapine	Moderate inhibition	CYP2D6	495,773
Venlafaxine	Quetiapine	Moderate inhibition	CYP2D6	469,792
Risperidone	Citalopram	Moderate inhibition	CYP2D6	395,110
Pantoprazole	Sertraline	Moderate inhibition	CYP2C19	378,066
Pantoprazole	Clopidogrel	Moderate inhibition	CYP2C19	359,023
Metoprolol	Citalopram	Moderate inhibition	CYP2D6	275,796
Citalopram	Esomeprazole	Moderate inhibition	CYP2C19	272,170
Risperidone	Quetiapine	Moderate inhibition	CYP2D6	268,359
Risperidone	Cholecalciferol	Moderate inhibition	CYP2D6	236,991
Metoprolol	Cholecalciferol	Moderate inhibition	CYP2D6	189,664
Risperidone	Venlafaxine	Moderate inhibition	CYP2D6	187,469

**Abbreviation**: PGx: pharmacogenetic.

**Table 5:** Top 15 Most Claimed Pairs of PGx Drugs and Inhibitors or Inducers Within ± 5 Days in the Five-Year Observation Period From 2017 to 2021

PGx drug	Inhibitor/ Inducer	Interaction Type	Enzyme	Number of Concomitant Claims
Pantoprazole	Quetiapine	Moderate inhibition	CYP2C19	545,255
Escitalopram	Quetiapine	Moderate inhibition	CYP2C19	140,172
Citalopram	Quetiapine	Moderate inhibition	CYP2C19	137,095
Metoprolol	Quetiapine	Moderate inhibition	CYP2D6	134,705
Venlafaxine	Quetiapine	Moderate inhibition	CYP2D6	100,819
Haloperidol	Quetiapine	Moderate inhibition	CYP2D6	95,966
Pantoprazole	Clopidogrel	Moderate inhibition	CYP2C19	89,312
Pantoprazole	Sertraline	Moderate inhibition	CYP2C19	78,168
Risperidone	Citalopram	Moderate inhibition	CYP2D6	73,790
Metoprolol	Cholecalciferol	Moderate inhibition	CYP2D6	60,093
Risperidone	Quetiapine	Moderate inhibition	CYP2D6	55,466
Metoprolol	Citalopram	Moderate inhibition	CYP2D6	54,819
Citalopram	Esomeprazole	Moderate inhibition	CYP2C19	53,553
risperidone	Cholecalciferol	Moderate inhibition	CYP2D6	48,041
Sertraline	Quetiapine	Moderate inhibition	CYP2C19	39,379

**Abbreviation**: PGx: pharmacogenetic.

## Discussion

Between 17.4% and 24.8% of individuals were exposed to potentially interacting drug pairs between 2017 and 2021. In addition, 1.5–2.2% of individuals were exposed to potentially strong interacting drug pairs. Individuals exposed to interacting drugs were frequently female, older and took more drugs than the general population. Interactions were more frequently associated with CYP2D6 or CYP2C19 than with CYP2C9. The drug pairs most frequently concomitantly used were pantoprazole-quetiapine, citalopram-quetiapine, metoprolol-quetiapine, and escitalopram-quetiapine. The results demonstrated that 70.3% of individuals had received at least one PGx drug. A preceding study, conducted using Helsana data from 2016 to 2020 and encompassing a greater number of genes, demonstrated that 74.7% of insured individuals had received at least one PGx substrate, indicating to a high prevalence of potential DGIs.^27^ In our study, 24.8%-35.3% of PGx drug users were concomitantly treated with an inhibitor or inducer of the respective metabolizing enzyme, indicating to a high prevalence of potential DDGIs. It is therefore essential to ensure that PGx test results are adequately assessed to prevent misclassification of the metabolizer phenotype of patients. Moreover, it can avert the subsequent administration of incorrect dosages or the inappropriate selection of a drug. Our study was limited to three enzymes, therefore the number of persons concomitantly using PGx drugs and inhibitors or inducers is likely even higher. However, Blagec et al investigated seven genes in a similar manner and found that 96% of all concomitant drug pairs detected were associated with the genes CYP2D6, CYP2C19, or CYP2C9.^18^ Therefore, it can be assumed that the selection of genes was appropriate. The disparate methodologies pertaining to patient populations, reference data, and the presence or absence of genetic data render a direct comparison of our work with other studies challenging. Furthermore, some studies evaluated the phenoconversion rate due to supplementary inhibitors or inducers, yet did not quantify these phenoconversions within a population.^33^,^34^ A study of Austrian claims data yielded comparable results, indicating that approximately one quarter of individuals prescribed a PGx drug were concurrently treated with an inhibitor or inducer of a drugs’ metabolism.^18^

Most drugs that were concomitantly used affect the nervous system. Quetiapine, escitalopram, citalopram, risperidone, haloperidol, and venlafaxine were particularly represented. A 2022 report by the Swiss Health Observatory revealed that psychotropic drugs constituted the most frequently prescribed medication group in Switzerland.^35^ This finding is clearly reflected in the results of our study. Studies investigating DDGIs in Denmark, Austria, the USA, or Australia, identified PPIs, clopidogrel, antidepressants, antipsychotics, and analgesics as the drugs most commonly involved.^2^,^18^,^34^,^36^ The list of the most interacting medications was created based on the ATC code. Consequently, certain active ingredients were represented in the rating on multiple occasions, either in combination with another active ingredient or in different pharmaceutical forms. Nevertheless, when these active ingredients are combined, there is no discernible alteration in the order of the most interacting medications.

Although the study population was almost balanced in terms of sex (52.4% women and 47.6% men), women had a higher prevalence of concomitant drug use than men (20.7% vs 13.9% (±5-days window)). The interaction pairs were analyzed, but no pair was identified as being particularly responsible for a high number of interactions and being taken exclusively by women. The proportion of gynecological drugs was found to be small. One potential explanation for the greater prevalence of concomitant drug use is that women tend to take a greater number of different drugs on average than men (23.3 vs 17.8 different drugs). This phenomenon has also been observed in other studies.^37^ Another potential explanation for the observed differences is that the women in the study population were, on average, older (46.1 vs 42.7 years).

The analysis was based on billing data from Helsana, which is subject to the limitations inherent in insurance claims data. Complete data on the use of OTC medications is unavailable. It should be noted that six PGx drugs (codeine, esomeprazole, flurbiprofen, ibuprofen, omeprazole, and pantoprazole) are available as OTC drugs in Switzerland, although they are also available on prescription. Of the inhibitors, clotrimazole, cholecalciferol, diphenhydramine, esomeprazole, ketoconazole, omeprazole, and terbinafine are available OTC. Cannabis is not available OTC, but the illegal procurement of this substance is not registered either. With regard to inducers, St. John’s wort is available OTC.^38^ Consequently, the prevalence of interactions with drugs available OTC is likely to be underestimated. Furthermore, before the healthcare insurance starts to reimburse claims, a small out-of-pocket payment on an annual basis must be made. This affects acute treatments more than chronic treatments, which could result in an underestimation of acute treatments.

The Helsana database does not contain any genetic data. Therefore, it was not possible to identify actual DDGIs or phenoconversions. To gain a deeper understanding of DDGIs, it is essential to utilize comprehensive data on drug utilization, in conjunction with an individual’s genetics, as exemplified by the UK Biobank.^39^

A further limitation of this study is that the exact pack sizes of the drugs were not considered. As the patients’ dosages are not specifically recorded in the database, even when the pack size is considered, it is not possible to determine the exact duration of a patient’s drug intake. The ±5-/30-days window was employed to analyze interactions in which the substrate was taken at the same time as an inhibitor or inducer. However, even when medications are claimed together, there is no certainty as to whether they were actually taken together. In contrast, some interactions may have been overlooked due to discrepancies in pack sizes. For chronic medications, pack sizes typically range between 30 and 100 tablets, which could extend beyond the defined time window.^38^

Despite the complexity of drug metabolisms, which include multiple enzymes and the impact of multiple morbidities and polypharmacy on patients,^40^,^41^ this study evaluated all interactions independently of one another. The concurrent administration of inhibitors and inducers of the same enzyme was not considered in the analysis. Furthermore, we did not investigate interactions between two substrates of the same enzyme, which could result in a competitive inhibition, a phenomenon that has been demonstrated to lead to phenoconversion.^42^ Furthermore, it is important to note that not all of the identified interactions necessarily result in phenoconversions. For instance, the phenotype of PMs remains unchanged, as non-functional proteins cannot be induced or inhibited.^3^

A strength of the study is the utilization of multiple sources for the determination of PGx drugs, inhibitors, and inducers. Moreover, the interactions were stratified according to the application route (systemically or locally) and the strength of the inhibitor or inducer. The distinction of ATC codes as either locally or systemically administered proved challenging due to the absence of differentiation in some ATC codes. This stratification was undertaken to assess the clinical relevance of the interactions. Furthermore, it could be employed to assess the priority of specific interactions for integration into a PGx clinical decision support system, with the aim of improving the prediction of a patient’s drug response phenotype.^18^

## Conclusion

This study highlights the necessity of considering non-genetic factors when interpreting PGx test results, given the high prevalence of the simultaneous use of PGx drugs with inhibitor and inducer drugs. The incorporation of DGIs and DDGIs into clinical practice and decision-making could facilitate the development of personalized drug therapies, thereby enhancing the safety and efficacy of treatments for patients. Clinical pharmacists are well placed to support this by identifying and managing DGIs and DDGIs, with the support of decision support systems that incorporate these elements. Consequently, further studies are needed to determine the prevalence and clinical significance of actual DDGIs. Future efforts should focus on validating the clinical impact of DDGIs in real-world settings, as well as developing decision support tools that synthesize genetic, pharmacological and clinical information.

## Data Availability

The datasets generated and/or analyzed during the current study are not publicly available due to confidentiality requirements issued by Helsana. Analysis codes and datasets can be made available by the corresponding author (s.allemann@unibas.ch) upon reasonable request and with permission of Helsana.

## Abbreviations

ADR, adverse drug reaction; ATC, Anatomical Therapeutic Chemical Classification System; CPIC, Clinical Pharmacogenetics Implementation Consortium; CYP, cytochrome P450; DDI, drug-drug interaction; DDGI, drug-drug-gene interaction; DGI, drug-gene interaction; DPWG, Dutch Pharmacogenetics Working Group; DRG, diagnosis-related group; FDA, US Food and Drug Administration; FOPH, Federal Office of Public Health; N, number of individuals; OTC, over-the-counter; PGx pharmacogenetic; PharmGKB, Pharmacogenetic Knowledgebase; PM, poor metabolizer; sd, standard deviation; TDM, therapeutic drug monitoring.
